# Research Progress of Porous Radiative Cooling Films Based on Phase Separation Method

**DOI:** 10.3390/nano16030190

**Published:** 2026-01-30

**Authors:** Shicheng Lu, Youliang Cheng, Mengyao Li, Jing Chen, Changqing Fang, Xingbo Yao, Changxue Cao, Jiamin Fan

**Affiliations:** 1 Faculty of Printing, Packaging Engineering and Digital Media Technology, Xi’an University of Technology, Xi’an 710048, China; 2 School of Mechanical and Precision Instrument Engineering, Xi’an University of Technology, Xi’an 710048, China; 3 School of Art and Design, Xi’an University of Technology, Xi’an 710048, China

**Keywords:** radiative cooling, phase separation, films, porous structure, thermal management

## Abstract

In recent years, against the backdrop of increasingly prominent global climate change and environmental issues, high-efficiency cooling technologies and energy-saving materials have become key research focuses. Radiative cooling, which reflects sunlight and emits thermal radiation into outer space, enables passive cooling without energy consumption. The phase separation method has emerged as a promising approach for fabricating porous daytime radiative cooling materials, attracting extensive research interest due to its favorable processability, excellent cooling performance, low cost, and scalability. Based on radiative cooling principles, this review summarizes the preparation methods, structural design, and application fields of porous radiative cooling films fabricated via the phase separation method. Furthermore, it is suggested that phase-separated porous radiative cooling films hold great potential in green buildings, personal thermal management, and food preservation.

## 1. Introduction

In recent years, the intensification of global warming, environmental degradation, and the increasing frequency of extreme weather events have posed severe challenges to sustainable development worldwide. The widespread adoption of conventional cooling technologies has led to a surge in energy consumption and greenhouse gas emissions, aggravating the vicious cycle of global warming [1]. By 2050, emissions attributable to building energy consumption are predicted to account for nearly 50% of the global total [2]. Consequently, to achieve environmentally friendly, efficient, and energy-saving solutions, the development of novel, green, high-efficiency, and energy-conserving cooling technologies has become an urgent research priority. Thermal radiation represents one of the most fundamental mechanisms of energy transfer. The universe, with its background temperature approaching absolute zero, constitutes both a vast renewable thermodynamic resource and an ultimate heat sink. Consequently, objects on Earth can dissipate heat into outer space via electromagnetic radiation through a process known as radiative cooling. High-temperature objects naturally emit thermal radiation to lower their temperature, while the atmospheric transparency window in the mid-infrared range of 8–13 µm enables objects at ambient temperature to radiate heat directly into the cold cosmos. Thus, thermal radiation can traverse the atmosphere and escape into space, where the temperature is approximately 3 K, making it theoretically possible for terrestrial objects to achieve sub-ambient cooling through radiative processes [1].

Passive daytime radiative cooling (PDRC) is an energy-free cooling technology that utilizes surface-engineered materials to achieve spontaneous and continuous cooling under direct sunlight. This is accomplished by strongly reflecting solar radiation (0.3–2.5 µm) while simultaneously emitting thermal radiation through the infrared atmospheric transparency window into outer space [3,4]. Owing to its notable advantage of operating without external energy input, PDRC has attracted considerable research interest. With recent advances in PDRC, a variety of novel structures and materials have been designed and fabricated to achieve high cooling performance, including nanoparticle-composite films [5], multilayer structures [6], and photonic crystals [7]. However, the practical application of such designs is often hindered by high costs and complex fabrication processes. In contrast, porous polymer films prepared via the phase separation method exhibit high solar reflectivity, low cost, and simple processing, making them a promising candidate for high-performance daytime radiative cooling materials.

Phase separation is a universal mechanism for the formation of biophotonic nanostructures, observed in the scales and bristles of various terrestrial arthropods such as beetles, butterflies, and bees [8]. The formation of these structures relies on the self-assembly of intracellular membrane systems, including the plasma membrane and endoplasmic reticulum. By regulating the curvature modulus of membrane-bound proteins, organisms can induce phase separation within lipid bilayers, forming specific lyotropic liquid crystal templates. Subsequently, chitin deposits and solidifies in the extracellular space, eventually yielding optically active nanostructures. The scales of the Cyphochilus beetle exemplify phase separation in creating highly scattering structures, where domain size can be controlled by adjusting the coarsening time of the separation process. Inspired by this natural design, Burg et al. [9] developed the first artificial biomimetic material using solvent-induced phase separation of cellulose acetate (CA), achieving a filling fraction of 21.5 ± 4% and a reflectance of 94%. Yang et al. [10] further extended this phase-separation approach through thermally induced phase separation to prepare polyethylene (PE) aerogels. With a high porosity of 97.9% and tailored pore sizes of 3.8 ± 1.4 μm, these aerogels demonstrate remarkable solar reflectance of 96% at just 2.7 mm thickness while maintaining a high mid-infrared transmittance of ~0.8. Combined with a low thermal conductivity of 0.032 W/(m·K), this cooling skin can achieve a daytime temperature reduction of 5–6 °C in urban environments, with a potential cooling limit of up to 14 °C under ideal conditions. Herein, this review begins with the principles of radiative cooling and summarizes recent progress in phase-separated porous radiative cooling films, focusing on their preparation methods, structural design, and application scenarios. Finally, future research directions and potential applications of phase-separated porous films are discussed.

## 2. Principles of Radiative Cooling

The cold universe serves as a natural and sustainable heat sink, analogous to the sun as a renewable energy source, providing the fundamental basis for passive heat dissipation from the Earth. To maintain the global thermal equilibrium, the planet continuously emits thermal radiation into space, balancing the solar energy it absorbs [11]. Radiative cooling technology leverages this heat balance principle by engineering the spectral properties of materials to exhibit high reflectivity in the solar spectrum (0.3–2.5 µm), thereby minimizing heat absorption, and high emissivity within the mid-infrared atmospheric window (8–13 µm), thereby enhancing thermal radiation emission toward outer space. When the power radiated from the surface exceeds the total heat gain from absorbed solar and atmospheric radiation plus convective and conductive heat exchange with the surroundings, a net cooling power is achieved. This enables the surface temperature to drop below the ambient level, realizing passive sub-ambient cooling without external energy input.

**Figure 1:**
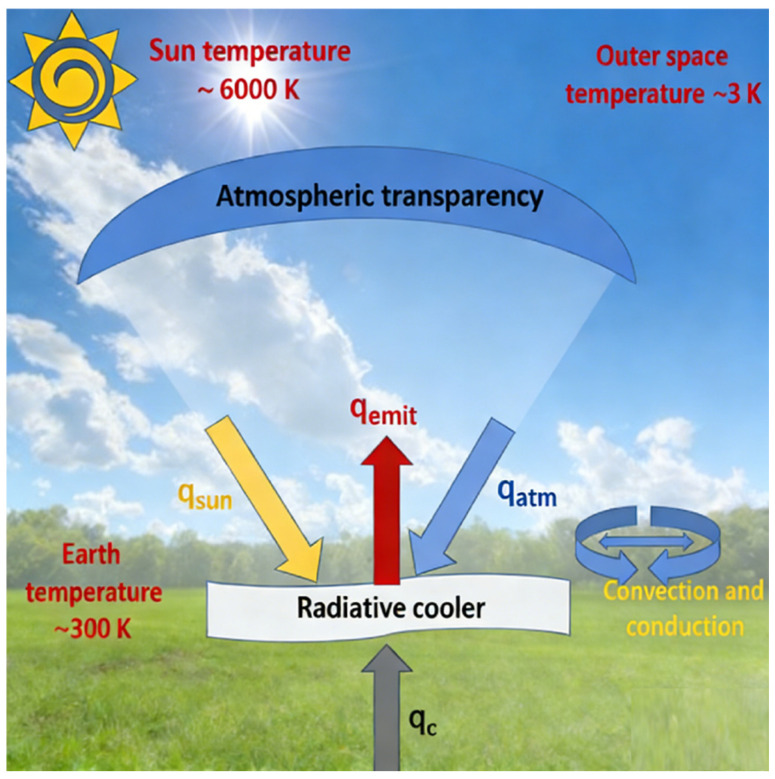
Radiative energy flow.

The cooling efficiency during radiative cooling is affected by heat flow [12], including heat radiation, heat conduction, and heat convection. The energy balance process during radiative cooling is shown in Figure 1, where q_emit_ is the radiated energy, q_sun_ is the absorbed solar energy, q_atm_ is the absorbed atmospheric radiation energy, and q_c_ is the inherent cooling loss.

The net cooling capacity of a material is determined by the combined effects of solar radiation, the material’s own thermal radiation, atmospheric thermal radiation, and non-radiative heat exchange. To clarify the relationship between radiative cooling performance and the emissive properties of the material surface, a system heat flow balance equation can be established. The net cooling power (*P*_cool_) can be expressed as Equation (1):

(1) $$P_{\mathrm{cool}}\;={\;P}_{\mathrm{emit}}\;-{\;P}_{\mathrm{atm}}\;-{\;P}_{\mathrm{sun}}\;-{\;P}_{c}$$

where *P*_cool_ is the net cooling power, *P*_emit_ is the output power of the radiative cooling material radiated externally, *P*_atm_ is the power received from the atmosphere, *P*_sun_ is the power of sunlight absorbed by the radiative cooling material, and *P*_c_ is the total power loss of all non-radiative heat transfer processes.

Additionally, the output power (*P*_emit_) of the radiative cooling material can be calculated using Equation (2):

(2) $$P_{\mathrm{emit}}\left(T_{s}\right)\;=\;A\int \cos\theta d\Omega \int_{0}^{\infty }I_{\mathrm{BB}}\left(T_{s},\lambda \right)\varepsilon \left(\lambda ,\theta \right)d\lambda$$

where A is the area of the radiator, *T*_s_ is the surface temperature of the radiator, $\varepsilon \left(\lambda ,\theta \right)$ represents the surface emissivity of the radiator at wavelength λ and angle of incidence θ, $I_{\mathrm{BB}}\left(T_{s},\;\lambda \right)\;$represents the power emitted per unit area, per unit solid angle, and per unit wavelength interval by an ideal blackbody at temperature *T*_s_ and wavelength *λ*.

*P*_atm_ is the power absorbed by the material from atmospheric radiation, calculated by Equation (3):

(3) $$P_{\mathrm{atm}}\left(T_{\mathrm{amb}}\right)\;=\;A\int \cos\theta d\Omega \int_{0}^{\infty }I_{\mathrm{BB}}\left(T_{\mathrm{amb}},\lambda \right)\varepsilon_{\mathrm{amb}}\left(\lambda ,\theta \right)\varepsilon \left(\lambda ,\theta \right)d\lambda$$

where $P_{\mathrm{atm}}\left(T_{\mathrm{amb}}\right)$ represents the radiant power received by the radiator from the atmosphere at a temperature of $T_{amb}$, $\varepsilon_{\mathrm{amb}}\left(\lambda ,\theta \right)=1\;-{\;t\left(\lambda \right)}^{1∕\cos\theta }$ is the spectral directional emissivity of the atmosphere, and $t\left(\lambda \right)$ is the atmospheric transmission coefficient at the zenith. $I_{\mathrm{BB}}\left(T_{\mathrm{amb}},\;\lambda \right)\;$represents the power emitted per unit area, per unit solid angle, and per unit wavelength interval by an ideal blackbody at temperature *T*_amb_ and wavelength *λ*.

*P*_sun_ is the power absorbed from solar radiation, calculated by Equation (4):

(4) $$P_{\mathrm{sun}}\;=\;A\cos\theta \int_{0}^{\infty }I_{\mathrm{sun}}\left(\lambda \right)\varepsilon \left(\lambda ,\theta \right)d\lambda$$

where *A* is the area of the radiator, $I_{\mathrm{sun}}\left(\lambda \right)$ can generally be selected from the solar spectrum irradiance of AM1.5, θ represents the angle between the normal direction of the radiating surface and a specific observation or radiation direction.

*P*_c_ is the power exchanged with the environment via conduction and convection, calculated by Equation (5):

(5) $$P_{c}\;=\;\mathrm{Ah}\left(T_{\mathrm{amb}}-T\right)$$

where *A* is the area of the radiator, *h* is the comprehensive coefficient describing this non-radiative heat transfer.

From the above balance relationship, it can be seen that the infrared radiative properties outside the atmospheric window are not fixed but are influenced by multiple factors such as the temperature of the cooled object, specific cooling requirements, and environmental conditions. For example, to achieve the lowest possible steady-state temperature in a radiative cooling device, heat input from the environment should be minimized, thereby reducing the radiative power P_emit_ required to maintain P_cool_ = 0. Therefore, an ideal device should exhibit low absorptivity and high reflectivity in bands outside the atmospheric window to maximize suppression of atmospheric background radiation absorption. Furthermore, by effectively blocking non-radiative heat exchange pathways such as conduction and convection, such devices can achieve temperature reductions exceeding 40 °C below the ambient temperature, demonstrating excellent thermal management performance [1].

## 3. Preparation of Radiative Cooling Films via Phase Separation

In recent years, researchers have successfully fabricated various high-performance daytime radiative cooling materials using different methods, such as photonic crystals [13,14,15], templating [16,17], electrospinning [18,19,20], blending [21,22], and phase separation [23,24,25,26]. Radiative cooling materials with periodic photonic structures face significant challenges in large-scale expansion due to their complex preparation processes and high costs. The templating method is limited in the large-area production of radiative cooling materials due to its intricate and delicate operations and reliance on template arrays, or it results in waste from the need for additional sacrificial raw materials as templates. Although electrospinning enables the large-area preparation of radiative cooling materials, the resulting fiber structures are often simplistic, and the process imposes stringent equipment requirements.

Phase separation is a simple and effective method for creating micro/nanopores. By inducing a micro/nanoporous structure within the polymer itself, it enables efficient reflection of sunlight, thereby avoiding the extensive use of micro/nanoparticles and significantly reducing raw material consumption. During phase separation, a polymer solution in a thermodynamically unstable state gradually tends toward thermodynamic stability, undergoing phase differentiation and ultimately forming two coexisting liquid phases. In this process, the polymer-rich phase solidifies to form a solid matrix, while the other phase, which is low in polymer concentration and rich in non-solvent, gradually creates a pore structure within the matrix [27]. Currently, phase separation techniques are categorized into various methods (as described in Table 1), including but not limited to thermally induced phase separation (TIPS), non-solvent induced phase separation (NIPS), vapor-induced phase separation (VIPS), evaporation-induced phase separation (EIPS), and reaction-induced phase separation (RIPS), and photopolymerization-induced phase separation (PIPS). Each of these methods relies on distinct physical or chemical mechanisms to achieve precise control and separation of phases in polymer systems, offering diverse pathways for the research and application of daytime radiative cooling materials [27].

### 3.1. TIPS

The core of TIPS lies in dissolving a polymer in a solvent with a high boiling point and low volatility at elevated temperatures to form a stable homogeneous solution. The system is then cooled to induce phase separation during the cooling stage. After the polymer solidifies, the diluent is removed, ultimately yielding the desired porous polymer film [28].

During the implementation of the TIPS process, the homogeneous polymer–solvent system undergoes liquid–liquid thermally induced phase separation upon cooling, leading to the formation and growth of diluent-rich droplets—a phenomenon referred to as coarsening. As the system continues to cool and solidify, the resulting film structure consists of a continuous polymer matrix interspersed with a network of solvent-occupied pores. Therefore, the size of the droplets directly determines the geometric dimensions of the pores in the final film, indicating a positive correlation between droplet size and pore size. Owing to this characteristic, the TIPS method is widely applied in the preparation of porous membranes from various polymers, including those that cannot form porous membranes via immersion precipitation due to solubility limitations. A typical example is polyolefins, which exhibit good heat and solvent resistance, low cost, and are suitable materials for porous films [27].

In 2022, Liu and co-workers [24] combined bio-inspired hierarchical structures with stereocomplex crystal design and prepared a micro/nanostructured polylactic acid (PLA) aerogel radiative cooler with a high stereocomplex content via a simple water-assisted TIPS technique. The PLA aerogel exhibited high reflectivity (89%) and high emissivity (87%), achieving passive daytime and nighttime temperature reductions of 3.5 °C and 5.8 °C, respectively. Moreover, as the stereocomplex content increased to 24%, the hierarchical micro/nanostructure imparted superhydrophobicity to the surface, with a water contact angle of 152°. The material also demonstrated high compressive strength (0.10 MPa) and low thermal conductivity (37 mW·m^−1^·K^−1^), along with excellent weather resistance, damage tolerance, and thermal stability. Zhou et al. [29] developed a method combining thermally induced phase separation with 3D printing technology to fabricate nanoporous polymer-based composites. By mixing high-density polyethylene, ultra-high molecular weight polyethylene, and SiO_2_ particles in paraffin oil at elevated temperatures to form a homogeneous solution, they induced solid–liquid phase separation through cooling after compression molding or 3D printing. This approach enabled the 3D printing of radiative cooling materials with complex structures, offering feasibility for large-scale applications.

**Figure 2:**
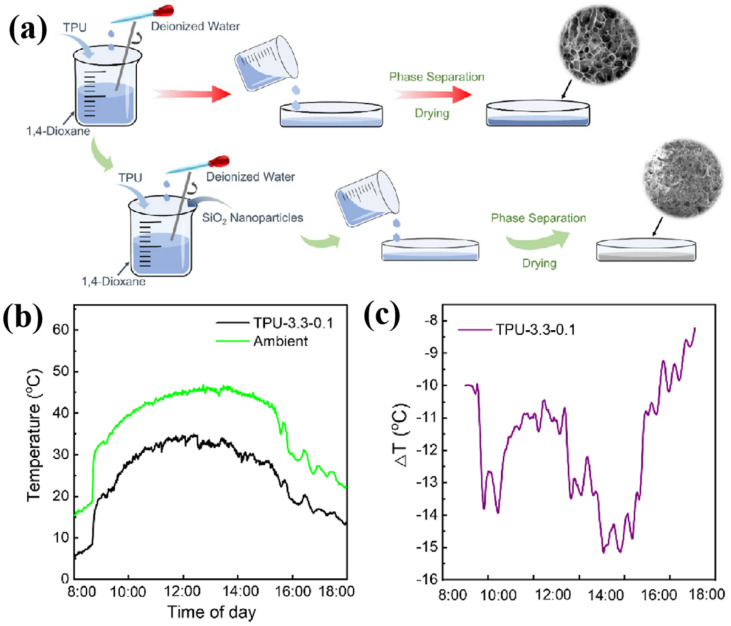
(**a**) Preparation processes of TPU and TPU/SiO_2_ foams using WA-TIPS method. (**b**) Temperature measurement of the sub-ambient cooling performance test of TPU−3.3−0.1 foam for daytime. (**c**) Extracted temperature difference for daytime [31].

In 2023, Yue et al. [30] successfully fabricated a dielectric/polymer composite film with a distinctive three-dimensional porous structure via thermally induced phase separation. The resulting composite film exhibits excellent spectral selectivity and efficient radiative cooling performance. Its internal structure consists of randomly distributed alumina particles and hierarchically disordered micro/nanopores. The rational hierarchical structure and functional components effectively enhance the spectral properties of the material, enabling strong solar reflectance (98.26%) and high infrared emissivity (97.56%). Under a solar irradiance of 890 W/m^2^ during a summer daytime, the film achieved an average cooling temperature of 9.1 °C below ambient, with an effective radiative cooling power of 87.2 W/m^2^. Additionally, Wang et al. [31] employed water-assisted thermally induced phase separation to prepare thermoplastic polyurethane foam with hierarchical micro/nanostructures (as shown in Figure 2a). As illustrated in Figure 2b,c, the surface temperature recorded on the foam remained significantly lower than the ambient temperature, with the maximum temperature reduction increased by nearly 650%.

### 3.2. NIPS

NIPS has emerged as a versatile and scalable technique for fabricating porous polymer membranes with tailored microstructures, demonstrating significant potential for radiative cooling applications. In a typical NIPS process, a polymer is first dissolved in an appropriate solvent to form a homogeneous solution. The subsequent introduction of a non-solvent, which is miscible with the solvent but immiscible with the polymer, triggers phase separation through solvent-non-solvent exchange. This process leads to the formation of a polymer-rich phase that solidifies into a continuous matrix and a polymer-lean phase that ultimately generates the porous structure upon solvent removal. The resulting pore morphology (including size, distribution, and connectivity) can be precisely tuned by adjusting parameters such as polymer concentration, solvent/non-solvent selection, and processing conditions [27,32]. The NIPS technique offers several distinct advantages for membrane fabrication including low production cost, convenient operation, hypotoxicity solvent systems, and controllable architectures of porous membranes. Owing to these benefits, NIPS has found widespread application not only in traditional fields such as filtration and separation, but also in emerging areas including energy materials, catalysis, and particularly radiative cooling.

**Figure 3:**
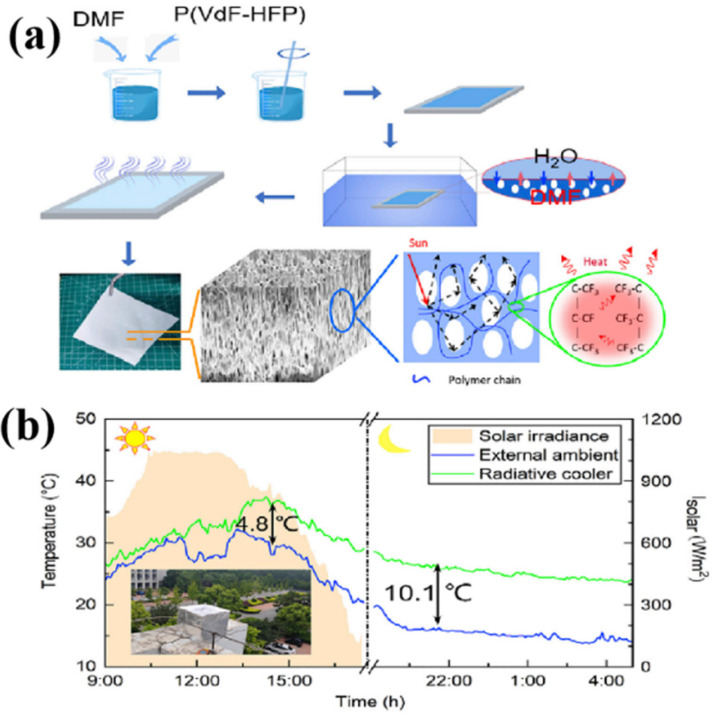
(**a**) The fabrication of TPC and its SEM image. (**b**) Schematic of the setup for testing performance of TPCs under sunlight and detailed solar intensity and temperature data on a clear but windy day in Tianjin [25].

As shown in Figure 3a, Zhu et al. [25] fabricated an anisotropic polymer radiative cooling coating using the NIPS process. When fully exposed to sunlight, this device achieved cooling up to 10.1 °C below ambient atmospheric temperature (in Figure 3b). Additionally, Kim et al. [33] reported the continuous fabrication of sponge-like polyimide films via NIPS, achieving a broad pore size distribution (0.1–10 μm) and high porosity (~80%). By systematically varying the non-solvent concentration during immersion curing, the authors demonstrated effective control over pore size, observing an inverse correlation between non-solvent content and pore dimensions. This work provided critical insights into the microstructural optimization of polyimide-based porous films for optical applications. Mandal et al. [34] employed NIPS in 2018 to prepare porous P(VDF-HFP) films. Using acetone as the solvent and water as the non-solvent, they deposited the polymer solution via spraying or drop-casting onto various substrates. The resulting films exhibited excellent optical properties, with a solar reflectance of 0.96 ± 0.03 and an atmospheric window emissivity of 0.97 ± 0.02, independent of the underlying substrate. Under direct solar irradiation, these films achieved a sub-ambient cooling effect of 6 °C and a cooling power of 96 W/m^2^. Finite-difference time-domain (FDTD) simulations revealed that the hierarchical pore structure (featuring pores around 0.2 μm and 5 μm) efficiently scattered visible and near-infrared light, respectively.

Further expanding the material palette, Xiang et al. [23] utilized water (non-solvent) and acetone (solvent) in a NIPS process to fabricate three-dimensional porous cellulose acetate (CA) films in 2021. A 40 μm thick layer of SiO_2_ microspheres was deposited beneath the CA film to enhance infrared emission, resulting in a solar reflectance of 97% and a mid-infrared emissivity of 95%, which is sufficient for effective daytime radiative cooling at a thickness of 150 μm. In a related study, Chen et al. [35] incorporated TiO_2_ nanoparticles into cellulose-based films via NIPS using anhydrous ethanol as the non-solvent. The inclusion of TiO_2_ boosted the solar reflectance to 97%. Scattering models attributed this high reflectance to random nanoparticle aggregation, which also contributed to enhanced mid-infrared emissivity. Radiative cooling tests confirmed a temperature reduction of approximately 10 °C below ambient under direct sunlight.

**Figure 4:**
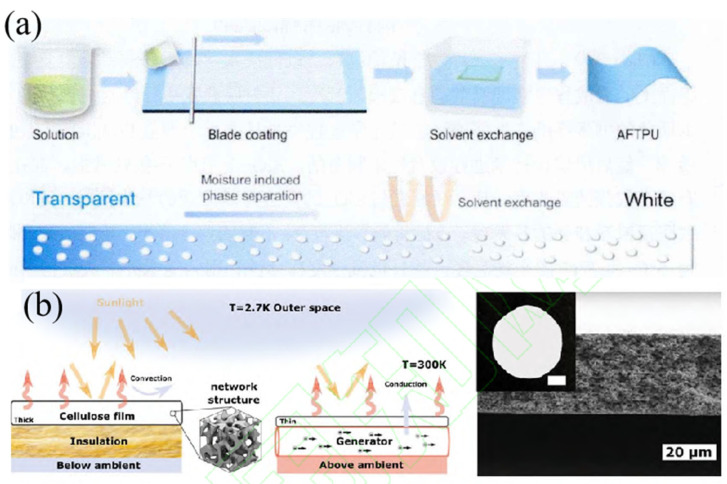
(**a**) Process diagram for preparing composite aerogel functionalized polyurethane film by non-solvent phase separation method [36]. (**b**) Scattering/radiation mechanism and microscopic morphology of porous cellulose radiative cooling film [37].

In 2022, Shan et al. [36] developed functionalized thermoplastic polyurethane (TPU) aerogel films using a scalable NIPS approach (in Figure 4a). The pure TPU films exhibited a solar reflectance of 94% and an atmospheric window emissivity exceeding 95%. By incorporating hydrophobic silica aerogel, the optical properties could be further tuned. The films demonstrated a sub-ambient cooling performance of 10 °C and a cooling power of 40 W/m^2^, alongside excellent water resistance and breathability, making them suitable for textile coatings. Also in 2022, Jaramillo-Fernandez et al. [37] prepared porous cellulose acetate films via solvent evaporation-induced phase separation (in Figure 4b). The films displayed a disordered porous structure that provided a solar spectrum reflectance of 95%. By varying the film thickness from 30 μm to 300 μm, the authors modulated the optical properties, achieving an atmospheric window emissivity of 93.6% and a daytime sub-ambient cooling effect of 7 °C.

Most recently, Cai et al. [38] designed a cellulose composite film (CCF) with a multi-level micro/nano structure through NIPS. The incorporation of ball-milled TiO_2_@potassium titanate as a dual-functional modifier significantly enhanced the mechanical properties (13-fold increase in Young’s modulus) and UV stability. The film achieved an ultra-high solar reflectance of 97.6%, attributable to its hierarchical pore architecture (micropores ~1 μm; nanopores ~300 nm). Importantly, the CCF maintained its cooling performance after 720 h of continuous UV exposure, demonstrating excellent durability. These studies collectively underscore the efficacy of NIPS as a powerful fabrication platform for designing polymer-based radiative cooling materials with tailored optical properties, mechanical robustness, and environmental stability. Liu et al. [39] combined microimprinting and phase separation techniques to fabricate a biomimetic photonic composite inspired by the microstructure of cicada wings. Microgroove arrays were first fabricated on a silicon template using photolithography, followed by spin-coating of a pre-solution consisting of thermoplastic polyurethane (TPU), alumina nanoparticles, and a solvent. The coated film was then immersed in a coagulation bath to undergo solvent–non-solvent-induced liquid–liquid phase separation, resulting in a polymer–ceramic composite film with micron-sized pores and surface microprotrusions.

### 3.3. VIPS

The pioneering work by Zsigmondy and Bachmann in 1918 introduced VIPS as a novel approach for solidifying homogeneous polymer solutions in vapor-phase environments [40]. This dry–wet-casting process involves exposing a polymer solution to a controlled vapor atmosphere, where the vapor evaporation under specific temperature and pressure conditions triggers phase separation through increased concentration of insoluble components. Although both non-solvent influx and solvent volatilization occur during VIPS, the non-solvent penetration dominates the phase separation mechanism. The method enables precise morphological control by regulating key parameters including exposure duration to humid air, relative humidity, and ambient temperature, offering significant potential for fabricating membranes with tailored structures for various applications [27].

Building upon this foundation, recent research has demonstrated the effectiveness of VIPS for developing high-performance radiative cooling materials. In 2023, Cheng et al. [41] employed a single-solvent VIPS approach to fabricate hierarchically porous PVDF-HFP fiber membranes with nanosphere structures. The selection of a UV-resistant PVDF-HFP polymer combined with the optimized VIPS process resulted in exceptional optical properties, achieving 93.7% solar reflectance and 91.9% infrared emissivity. The membranes demonstrated a remarkable temperature reduction of 13.2 °C compared to cotton fabric-covered skin, while the inherent UV resistance of the polymer matrix ensures enhanced durability for long-term radiative cooling applications in textiles. Zou et al. [42] developed a one-step water-vapor-induced phase separation method, wherein a concentrated PS solution in DMF was spin-coated in a high-humidity environment (e.g., 95% relative humidity). The rapid infiltration of water vapor into the liquid film triggered spinodal decomposition of the polymer solution, leading to the formation of a bicontinuous nanoporous structure. By precisely controlling spin-coating speed, solution volume, and humidity, the thickness (ranging from 3.5 to 84 μm) and pore morphology of the films could be accurately regulated.

When compared to other phase separation methods, VIPS offers distinct advantages for radiative cooling applications. Unlike TIPS, which requires precise temperature control, or NIPS, which involves liquid non-solvent immersion, VIPS provides gentler phase separation conditions through vapor–phase interactions. This characteristic enables better control over pore structure formation and distribution, particularly valuable for creating the hierarchical porous structures essential for efficient radiative cooling. The vapor-based approach also minimizes material waste and reduces environmental impact, making it suitable for scalable production of advanced radiative cooling membranes.

### 3.4. EIPS

As a dry-casting process, EIPS was first proposed by Kesting in 1973. In EIPS, polymers are dissolved in a specially formulated solvent system containing high-concentration solvents and multiple non-solvent components. As the volatile solvents gradually evaporate, the less volatile non-solvent components coalesce into fine droplets, whose stable presence provides sufficient time for polymer solidification. Finally, by removing these non-solvent-rich droplets, a porous structure is formed within the polymer matrix [27].

In 2023, Wu et al. [43] introduced EIPS prior to immersing the casting solution into the coagulation bath to regulate the mass transfer rate between the solvent and non-solvent during NIPS, thereby delaying the phase transition process. By controlling the EIPS duration and ambient temperature and humidity conditions, they successfully tailored the microstructure and overall properties of polysulfone membranes. Experimental results demonstrated that with increased pre-evaporation time, the membrane pore morphology transitioned significantly from a typical finger-like pore structure to a more uniform cellular-like spherical pore arrangement.

Building on conventional EIPS, a novel foam phase separation method has been developed by incorporating continuous feeding and stirring to generate foam, enabling rapid solvent evaporation and phase separation within the foam phase [44]. This technique, characterized by rapid reaction, high yield, and continuous production capability, shows potential as a scalable process for manufacturing porous polymer membranes [45,46].

### 3.5. RIPS

RIPS begins by uniformly mixing a solvent with a pre-polymer to form a homogeneous system prior to polymer curing. As the polymerization reaction proceeds, the molecular chains of the polymer gradually extend, accompanied by dynamic changes in intermolecular interactions and conversion rates resulting from chemical modifications. This series of changes ultimately leads to phase separation within the system. Subsequently, by removing the solvent, a porous polymer film is successfully constructed [47]. Naga et al. [48] employed the RIPS method to synthesize a porous material featuring a three-dimensional bicontinuous interconnected pore structure. By adjusting the reaction conditions and phase separation parameters, they were able to regulate the three-dimensional architecture of the material. It was ultimately concluded that the morphological structure of the polymer material is determined by the relative rates of the reaction and phase separation processes.

### 3.6. PIPS

PIPS begins with a homogeneous mixture of a photosensitive pre-polymer and a solvent. Upon exposure to light, the photoinitiated polymerization drives the extension of polymer molecular chains, accompanied by dynamic changes in crosslinking density, solvent–polymer compatibility, and conversion rates [49,50]. These transformations lead to controlled phase separation within the system. Subsequent removal of the solvent yields a structured porous polymer film. Luo et al. [51] employed PIPS to prepare coatings using trimethylolpropane triacrylate and vinyltriethoxysilane (VTES) as monomers, with VTES serving simultaneously as the porogen. Under UV irradiation, photopolymerization-induced phase separation was triggered, leading to the formation of interconnected nano- to micron-sized pore channels. By optimizing the monomer ratio and UV exposure time, the coating could be cured within seconds at room temperature. The resulting coating exhibited superhydrophobicity without requiring any post-treatment and could be fabricated over large areas through techniques such as blade coating. It has been demonstrated that the final morphology of the polymer is governed by the competition between the photopolymerization kinetics and the phase separation dynamics.

## 4. Design of Porous Radiative Cooling Films Based on Phase Separation Method

High solar reflectance and high mid-infrared emissivity are prerequisites for materials to achieve high-performance daytime radiative cooling [52,53]. Through appropriate material selection, a controlled membrane structure, and rational particle incorporation, porous films based on phase separation methods can realize effective daytime radiative cooling. Proper selection of the matrix material is essential to ensure radiative cooling performance. Rational control of the membrane structure can further optimize cooling efficiency. The addition of particles with high solar reflectance and high mid-infrared emissivity can additionally enhance the overall radiative cooling performance [54].

### 4.1. Selection of Substrate Materials

**Figure 5:**
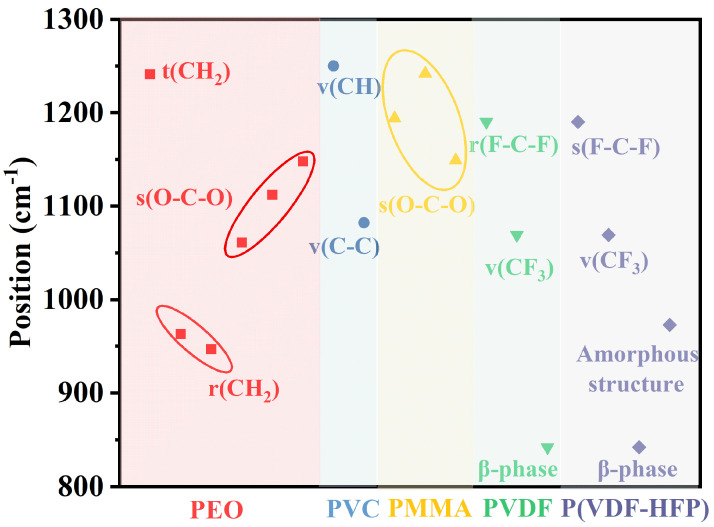
Vibration modes and vibration regions of functional groups of polymers commonly used to make porous membranes.

The substrate material serves as the foundation of porous radiative cooling films, with ideal candidates exhibiting excellent optical properties, thermal stability, and mechanical strength. Polymeric materials are particularly suitable due to their favorable gelation properties, which facilitate film formation via methods such as flow coating or doctor blading in phase separation processes. The resulting films often demonstrate superior mechanical strength, heat resistance, and chemical stability. In structural characterization, spectroscopic techniques, especially infrared spectroscopy, are widely employed. The region from 4000 to 1300 cm^−1^ is commonly referred to as the functional group region, as absorption peaks in this range primarily arise from stretching vibrations of specific functional groups, offering high identifiability. The region from 1300 to 400 cm^−1^ is termed the fingerprint zone due to the dense clustering of absorption peaks. The overlap between the fingerprint region and the atmospheric window (8–13 μm, corresponding to 1250–769 cm^−1^) provides a critical basis for selecting substrate materials in phase separation methods [55,56]. Polymers containing functional groups that vibrate within the fingerprint region tend to exhibit high mid-infrared emissivity within the atmospheric window [57]. Commonly used polymers in radiative cooling applications include PEO, PVC, PMMA, PVDF, and P(VDF-HFP), among others. Figure 5 summarizes the vibrational modes and corresponding spectral regions of functional groups present in these polymers, which are frequently utilized in the fabrication of porous membranes. However, certain fluorinated polymers, particularly PVDF and its copolymer P(VDF-HFP), have raised significant concerns due to their potential to cause endocrine disruption and immune system effects. While the environmental stability of these PFAS polymers benefits material performance, it complicates their end-of-life management and contributes to long-term ecological pollution.

### 4.2. Solvent Selection

In the membrane fabrication process, solvent selection critically influences polymer solubility and dissolution kinetics. An appropriate solvent ensures complete polymer dissolution and formation of a homogeneous solution, which is essential for subsequent film formation. Key solvent properties, including polarity, molecular weight, and viscosity, govern polymer chain conformation and intermolecular interactions in solution, thereby dictating the ultimate microstructure and performance of the resulting film. Solvents with strong dissolution capacity enhance the mobility of polymer chains, allowing them to adopt an extended conformation. In contrast, poor solvents tend to induce chain aggregation and coiling. The use of highly polar solvents, such as DMAc, DMF, and DMSO, accelerates phase separation rates, yielding films with minimal shrinkage, larger micropores, and higher water flux. Notably, among these solvents, DMSO offers the advantages of relatively lower toxicity and reduced emissions of volatile organic compounds (VOCs) compared to DMF, making it a more environmentally friendly and safer option in processing [58]. Conversely, low-polarity solvents slow the dual-diffusion process, resulting in compressed micropore morphology but generally enhanced mechanical strength of the final membrane.

Table 2 summarizes commonly used solvents and their solubility parameters, providing a practical reference for rational solvent design in phase separation-based membrane fabrication. The choice of solvent system significantly affects the comprehensive performance of polymeric membranes, including their mechanical properties, permeability, and chemical stability. For instance, membranes fabricated using strong polar solvents often exhibit enhanced hydrophilicity and superior permeability but may demonstrate relatively lower chemical stability. In contrast, films prepared with non-polar solvents generally possess improved chemical resistance and hydrophobic characteristics, albeit potentially at the expense of permeability. A representative study was conducted by Fang et al. [59], in which flat-sheet and hollow-fiber nanofiltration membranes were prepared through the NIPS method using polyethersulfone (PES) and sulfonated polyethersulfone (SPES) blends. Experimental results demonstrated that membranes fabricated with DMAc as the solvent achieved optimal separation performance, showing simultaneously high-water flux and rejection rates. This suggests that the strong polarity of DMAc facilitates the formation of an appropriate microporous structure during the phase separation process, thereby enhancing the membrane’s separation efficiency.

### 4.3. Selection of Nanoparticles

The incorporation of nanoparticles offers a promising pathway to further improve the performance of porous radiative cooling films [67]. These nanoparticles can selectively absorb or reflect radiation in specific wavelength ranges, enabling precise modulation of both solar and atmospheric window bands [68,69]. Commonly employed nanoparticles include TiO_2_ [35,70], SiO_2_ [71], Al_2_O_3_ [72,73,74], and BaSO_4_ [75]. By carefully selecting nanoparticle type and size, the optical properties of the composite films can be optimized to enhance solar reflectance and atmospheric window emissivity, thereby significantly improving the overall radiative cooling performance.

#### 4.3.1. Selection of Nanoparticle Size

The size of nanoparticles significantly influences their spectral radiative properties, thereby affecting the overall cooling performance of the composite films. Within specific spectral ranges (e.g., 0.2–26 μm), nanoparticles of different diameters exhibit distinct reflectance and emissivity behaviors. According to Mie scattering theory, appropriate selection of nanoparticle diameter can maximize the scattering of solar radiation, thereby enhancing the solar reflectance of the film [76].

Most studies determine the optimal particle size by evaluating the spectral radiative characteristics of particles with different dimensions. Yu et al. [77] embedded hollow yttria spheres (HYS) into a PDMS matrix, where FDTD simulations and experimental verification demonstrated the role of HYS in enhancing solar reflectance and long-wave infrared emissivity, as well as validating the effect of particle surface morphology on light scattering efficiency. In the work of Song et al. [78], three commercial TiO_2_ pigments were thoroughly characterized for their particle size distributions, and the correlation between size distribution features and optical performance was investigated. The results indicated that Altiris 550 and Altiris 800 showed more pronounced growth in geometric mean particle size and broader size distributions compared to Ti-Pure R-902. Miao et al. [79] studied the influence of Si_3_N_4_ particles with diameters of 1.96, 0.96, 0.71, and 0.45 μm on the spectral radiative properties of PU/Si_3_N_4_ composite films. They found that the solar reflectance of the film increased as the particle size decreased, with the film containing 0.45 μm Si_3_N_4_ particles achieving the highest solar reflectance. This insight deepens the understanding of the relationship between the microstructure of radiative cooling materials and their spectral properties, providing important guidance for the design of high-performance cooling films.

Variations in nanoparticle size alter their optical properties, including their scattering, absorption, and emissivity characteristics, which in turn affect the film’s surface emissivity and its capacity to emit infrared radiation into outer space. Appropriately sized nanoparticles can enhance the emissivity within certain wavelength ranges, thereby improving cooling efficiency. Therefore, the size and optical properties of nanoparticles must be carefully considered in the design and preparation of nanoparticle-embedded radiative cooling films to achieve optimal performance. Furthermore, by tuning the size and distribution of nanoparticles, the cooling effect can be effectively regulated and optimized.

#### 4.3.2. Mass Fraction of Added Nanoparticles

**Figure 6:**
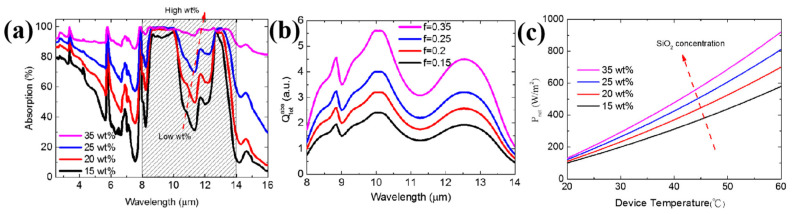
(**a**) Absorption spectra of the SiO_2_-PMMA nanocomposite film (**b**) resultant powers of collective incoherent SiO_2_ particles, and (**c**) effect of SiO_2_ content on film net radiant power [80].

The mass fraction of nanoparticles embedded in composite films is a critical factor influencing material cost, solar reflectance, and infrared emissivity, thereby directly determining the overall radiative cooling performance. Lee et al. [80] investigated the effect of SiO_2_ content on the net radiative power (Pnet) of PMMA/SiO_2_ composite films (as shown in Figure 6). The results showed that the net radiative power increased approximately linearly with rising SiO_2_ concentration, and radiative cooling efficiency was optimized by adjusting the SiO_2_ content. A system with 35 wt% SiO_2_ was able to capture more absorbed power within the transparent atmospheric window. In addition, the design of radiative cooling films based on hollow SiO_2_ microspheres (including both raspberry-like hierarchical structures [81] and uniformly dispersed single configurations [82]) follows a common strategic principle. It is the synergistic optimization of spectral selectivity through the structural modulation of internal cavities and surface morphologies. The hollow structures, combined with shell materials such as SiO_2_, create refractive index gradients that strongly enhance solar reflectance via multiple scattering. Meanwhile, SiO_2_’s intrinsic phonon resonance in the 8–13 μm atmospheric window, together with the high radiation surface area provided by the microspheres, ensures high thermal emittance in the long-wave infrared. As a result, these films demonstrate significant sub-ambient cooling in outdoor testing and present promising feasibility for scalable fabrication through compounding with polymer matrices such as PDMS.

The rational incorporation of nanoparticles plays a key role in optimizing the radiative cooling performance of composite films. An appropriate mass fraction can effectively enhance both solar reflectance and thermal emissivity, leading to improved cooling efficiency. However, excessive nanoparticle loading may not only diminish optical performance due to agglomeration effects but also increase production costs and compromise mechanical integrity. Therefore, the optimization of the nanoparticle mass fraction should be carefully tailored according to the specific material system, balancing cooling efficiency, cost-effectiveness, and practical feasibility to achieve an optimal performance-to-cost ratio.

### 4.4. Porous Structure

In the exploration of polymer microstructure design, researchers have identified a unique porous architecture that exhibits optical characteristics analogous to those of embedded nanoparticles. Such porous structures can achieve low solar absorption and high mid-infrared emissivity. Notably, the scattering efficiency of porous polymers in the solar spectrum is closely linked to their internal pore size distribution [83]. Based on Mie scattering theory, the scattering efficiency of a single-pore-size polymer is significantly influenced by pore diameter: as the pore size increases, the scattering efficiency gradually decreases, and the peak scattering wavelength undergoes a corresponding red shift [34]. Further studies have revealed that the infrared emissivity of single-pore-size polymers initially increases and then decreases with increasing pore size, though the overall variation remains relatively modest [83].

**Figure 7:**
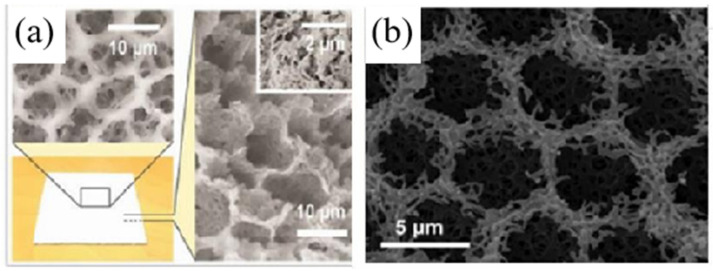
The morphology of radiator with porous structure under different magnifications: porous structure under (**a**) low and (**b**) high magnification [24].

Mandal et al. [70] fabricated a hierarchically porous PVDF-HFP film via a phase-inversion method (in Figure 7). The resulting film exhibited substrate-independent high solar reflectance (0.96 ± 0.03) and high atmospheric window emissivity (0.97 ± 0.02). Under direct solar irradiation, the film achieved a sub-ambient temperature reduction of 6 °C and a cooling power of 96 W/m^2^. Experimental and FDTD simulation results indicated that pores around 0.2 μm and 5 μm effectively scatter visible and near-infrared light, respectively.

**Figure 8:**
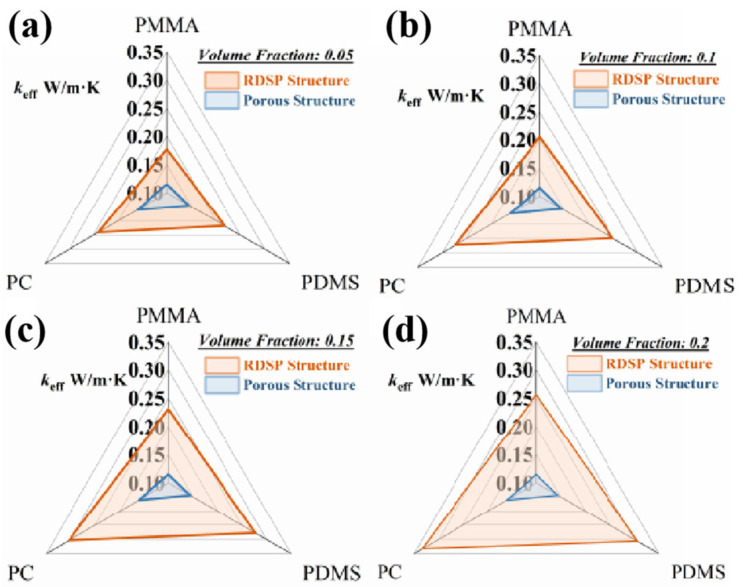
Thermal conductivity of radiative cooling film for different structures: (**a**) fv = 0.05, (**b**) fv = 0.1, (**c**) fv = 0.15, and (**d**) fv = 0.2 [84].

As shown in Figure 8. Du et al. [84] systematically studied the influence of parameters (diameter D, volume fraction fv, and thickness t) in random-distribution structures and porous structures on the radiative properties of common plastic materials. It was found that under conditions of significant indoor–outdoor temperature differences, films employing polymer porous structures can effectively block external environmental heat, thereby maintaining their cooling performance. Through numerical modeling, Chen et al. [83] systematically analyzed the relationship between pore size and scattering efficiency. Increasing pore size enhances the scattering efficiency within the corresponding wavelength band. Pores primarily sized at 100 nm and 200 nm can effectively scatter the full spectrum of sunlight, yielding higher solar reflectance. Instead, infrared emissivity is closely correlated with the effective thickness of the polymer. Below 200 μm, emissivity increases rapidly with thickness and stabilizes when the thickness exceeds 200 μm. In summary, the design and application of such porous polymers require comprehensive consideration of the pore size distribution to optimize both solar reflection and mid-infrared emission performance.

## 5. Application Prospects

Radiative cooling films represent an emerging class of passive cooling materials characterized by zero energy consumption and environmental friendliness. By efficiently lowering temperatures through radiative heat exchange, these films hold significant application potential in fields such as building energy efficiency, personal thermal management, and food preservation.

### 5.1. Building Construction

Among various sectors, the building industry is a major energy consumer. It is estimated that buildings account for over 40% of global energy consumption annually, with air conditioning systems contributing to approximately 65% of this share [85,86].

**Figure 9:**
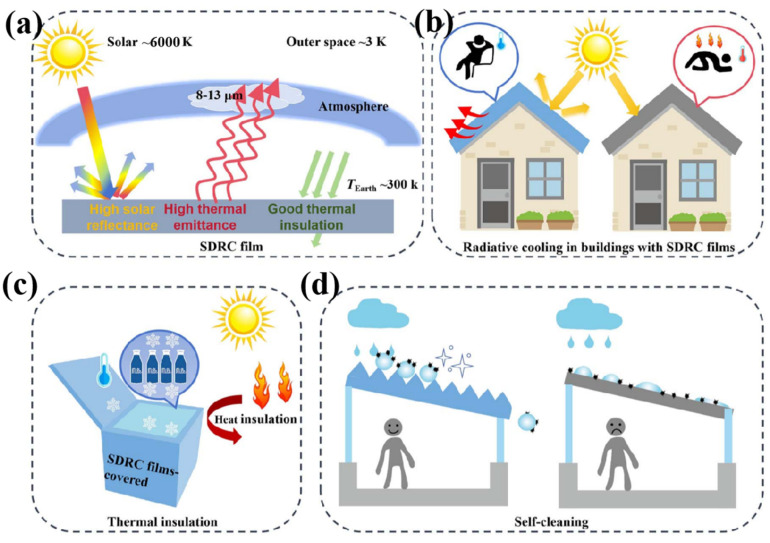
Schematic illustration and design of the SDRC film with multifunctional properties and its fabrication [90]. (**a**) Working principle of the SDRC film. (**b**) Application of SDRC as a roofing material for building energy savings. (**c**) Thermal insulation performance of the SDRC film, demonstrating its heat resistance. (**d**) Superhydrophobic self-cleaning properties of the SDRC film.

Radiative cooling films, when integrated into building surfaces such as roofs, can significantly lower indoor temperatures by dissipating heat into outer space [87,88]. This capability reduces reliance on conventional cooling systems, thereby decreasing energy consumption and carbon emissions while supporting environmental sustainability. Currently, roof integration represents the primary architectural application of these materials [89]. As shown in Figure 9, Tan et al. [90] developed a film with hierarchical micro/nanostructures composed of porous PDMS, micron-sized glass microspheres, and hydrophobic silica nanoparticles. This film achieved a solar reflectance of 94.2% and an infrared emissivity of 95.6%. The synergistic design enabled a sub-ambient temperature reduction of up to 7.8 °C under direct sunlight. Moreover, the super-hydrophobic self-cleaning property effectively prevents surface contamination and wetting, ensuring long-term operational stability and efficiency.

Li et al. [91] optimized a PVDF-HFP porous membrane prepared by the NIPS process, achieving sub-ambient cooling of approximately 5.4 °C during daytime (under a solar irradiance of 945 W·m^−2^) and up to 11.2 °C at night. This enables the material to address diurnal temperature variations and maintain continuous cooling performance. Jia et al. [92] developed a poly(lactic acid) (PLA) aerogel via TIPS, and for the first time quantified its energy-saving potential for building cooling systems across different cities: annual energy savings of 2.2–10.2 mJ·m^−2^, corresponding to an energy-saving rate of 8.2–24.3%. The dual function of “thermal insulation + radiative cooling” makes it particularly suitable for building envelope applications, as it can simultaneously reflect solar radiation and block heat conduction. Ma et al. [93] integrated passive radiative cooling materials with curtains, creating smart curtains that also enable dynamic, on-demand thermal management. Outdoor tests demonstrated that, compared to pristine cotton fabric, the material achieved an average daytime cooling of 10.3 °C while exhibiting excellent thermal insulation at night. This work extends the application of radiative cooling materials from static building components (e.g., walls, roofs) to movable and adjustable curtain systems, thereby allowing responsive thermal regulation.

Besides the phase separation method, radiative cooling films prepared by other methods can also be used for building cooling applications. For instance, Jung et al. [94] developed a micro/nanoporous poly(vinylidene fluoride-co-hexafluoropropylene) (PVDF-HFP) film. This film achieved a solar reflectance of 0.96 and an atmospheric window emissivity of 0.97, yielding a sub-ambient cooling effect of approximately 6 °C. Importantly, the film can be applied like a coating onto various substrates, making it suitable for diverse surfaces such as vehicle roofs and building facades. Feng et al. [95] designed a bilayer porous polymer membrane consisting of a hygroscopic hydrogel layer and a hydrophobic top layer with hierarchical pores. The hydrogel enables daytime evaporative cooling and nighttime self-regeneration, achieving a sub-ambient temperature reduction of about 7 °C. This system offers great potential for low-cost, efficient, and scalable passive building cooling.

While numerous experimental models have validated the cooling performance of radiative cooling films, large-scale building applications face challenges related to fabrication, installation, and maintenance complexity. Furthermore, these films exhibit excellent thermal stability, hydrophobicity, and flexibility to adapt to diverse architectural and climatic conditions, alongside long-term durability and anti-aging performance. In summary, radiative cooling films hold considerable promise for building energy conservation, yet their broad adoption requires overcoming practical challenges and continued exploration of novel material and integration strategies.

### 5.2. Personal Thermal Management

In recent years, global warming has intensified heat stress, posing serious threats to daily life and public health. While traditional cooling devices such as air conditioners can effectively reduce indoor temperatures and provide comfortable living and working environments, they suffer from high energy consumption and are often impractical for outdoor use due to economic and operational constraints [96]. Radiative cooling technology, characterized by zero energy consumption and environmental friendliness, has emerged as a promising solution for personal thermal management. By designing textiles with tailored spectral emissivity, it is possible to efficiently dissipate excess body heat and enhance human thermal comfort.

**Figure 10:**
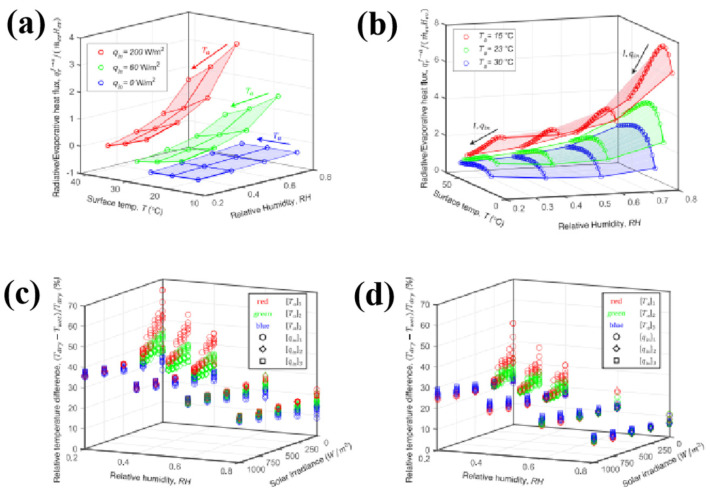
Cooling performance of a PE fabric for personal passive thermal management at different ambient conditions [97]. (**a**) Analysis of the ratio between the radiative and evaporative heat fluxes from the fabric top surface in indoor conditions. (**b**) Analysis of the ratio between the radiative and evaporative heat fluxes from the fabric top surface in outdoor conditions for different environmental parameters. (**c**) Ratio between the surface temperature in dry and wet outdoor conditions for the considered woven PE fabric and (**d**) for an ideal fabric with *τf* = 1.

As shown in Figure 10, Alberghini et al. [97] applied hydrophilic porous materials for the evaluation of personal thermal management performance. High relative humidity and low ambient temperature lead to low evaporation rates and enhanced infrared radiation, thereby favoring radiative heat transfer over evaporative heat transfer. Under conditions of minimal evaporation rate, the relative temperature difference consistently remained within 20% and gradually increased as the relative humidity decreased. The maximum relative difference (with a peak around 67%) occurred under lower ambient temperature, relative humidity, specific input heat flux, and solar irradiance conditions. He et al. [98] employed a NIPS method to fabricate a bionic white-fungus-inspired porous polyurethane (PU) membrane. This demonstrates the feasibility of integrating the porous PU membrane with textiles to form hierarchically porous fabrics. After undergoing abrasion, acid–base, and UV aging tests, the material retained high reflectivity, exhibiting excellent mechanical stability and environmental durability. Hydrophobic modification further endowed the material with self-cleaning capabilities. Further advancing this field, Yang et al. [99] developed a trilayer gradient structure consisting of a hydrophobic outer layer (SiO_2_/PVDF-HFP) and a hydrophilic inner layer (cotton) via a one-step phase separation method. This design, for the first time, integrates both radiative cooling (passive temperature reduction) and unidirectional moisture transport (active sweat wicking) within a single textile. The material exhibits a sweat evaporation rate of 0.029 g·m^−2^·s^−1^ and a reduced evaporation enthalpy of 2084 J/g. The hydrophilic cotton inner layer, in direct contact with the skin, provides a soft and comfortable feel, while the hydrophobic outer layer offers protective functionality without compromising breathability. This structural configuration effectively balances performance and comfort, making it particularly suitable for everyday wearable applications.

Beyond phase separation techniques, other fabrication methods have also achieved significant progress in the field of personal thermal management textiles. Cai et al. [100] developed a nanocomposite textile with selective spectral properties for outdoor personal thermal regulation. The fabric consists of zinc oxide (ZnO) nanoparticles embedded in a porous polyethylene (PE) matrix, exhibiting over 90% solar reflectance and selective emissivity within the atmospheric window. Experimental results demonstrated that the textile could lower simulated skin temperature by 5–13 °C compared to conventional fabrics. Miao et al. [101] coated one side of a commercial cotton substrate (single-faced knit, 170 g·m^−2^, thickness 500 μm) with a cellulose acetate (CA) solution (acetone/water) to form a porous CA layer approximately 50 μm thick, while the reverse side was treated with a hydrophobic emulsion (FCB015). The resulting fabric achieved a solar reflectance of 93.4% and a mid-infrared emissivity of 96.3%, reducing skin surface temperature by about 4.2 °C when worn.

Although these advanced textiles exhibit superior radiative cooling performance compared to conventional fabrics, comprehensive evaluations of other functional properties, such as breathability, moisture absorption, flexibility, and mechanical durability, remain limited. Future studies should systematically assess these parameters to ensure practical comfort and long-term usability without compromising cooling efficiency. Additionally, aesthetic attributes including color, texture, and visual appearance warrant further investigation to improve market acceptance and practicality.

### 5.3. Ice and Snow Protection/Food Preservation

Global warming has led to glacier melting and rising sea levels, exerting profound impacts on both ecosystems and human society. Radiative cooling materials, which enable temperature reduction through radiative heat exchange, offer a promising approach to address these challenges. When applied to ice and snow surfaces, these materials can lower temperatures below the melting point, effectively delaying thawing processes. Furthermore, radiative cooling technology holds significant potential in food preservation. The cold chain logistics, involving storage, transportation, and retail, are energy-intensive, and growing demand for high-quality food has escalated energy consumption, imposing substantial pressure on resources and the environment [102]. Approximately 14% of global food production is lost between harvest and retail due to inadequate refrigeration and power shortages [103]. Such waste not only represents economic loss but also contributes to greenhouse gas emissions from decomposition. Implementing radiative cooling materials within the food cold chain can mitigate resource constraints and reduce environmental burdens.

Covering ice or food items with radiative cooling films can effectively extend their preservation period. Li et al. [104] developed a durable hybrid metamaterial (DHM) via a straightforward phase separation method. The DHM achieves a solar reflectivity of 98.0% and a mid-infrared emissivity of 98.2%, which enables a significant reduction in surface temperature (averaging 9.0 °C) under solar illumination, thereby effectively slowing the melting rate of ice and snow. The high electronegativity of the C-F bonds in PVDF-HFP and the exceptional thermal and chemical stability of zirconium dioxide microparticles (ZrO_2_ MPs) endow the DHM with outstanding weatherability, chemical resistance, and mechanical durability. Compared to conventional ice and snow protection methods, such as salt spreading, mechanical removal, and artificial shading, the passive radiative cooling mechanism of the DHM offers advantages including zero energy consumption, pollution-free operation, and long-term sustainability. In addition, the DHM exhibits superior environmental durability over other radiative cooling materials, making it particularly suitable for prolonged outdoor applications.

**Figure 11:**
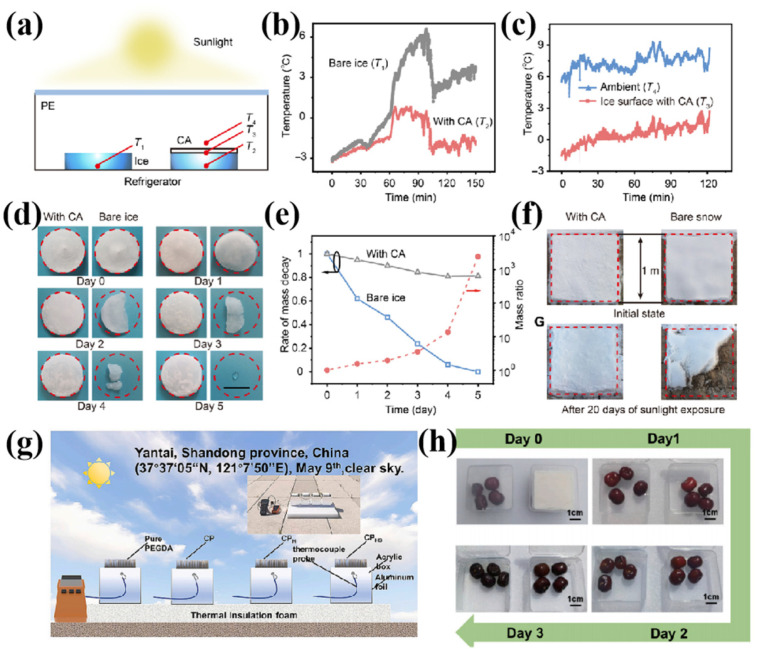
Ice and snow protection via the hierarchically designed CA film. (**a**) A schematic of the custom-made device for verifying the cooling effects of the hierarchically designed CA film for ice. (**b**) Comparison of the temperature of ice with and without the hierarchically designed CA film. (**c**) Sub-ambient cooling enabled by the hierarchically designed film under intense illumination of sunlight of ~700 W·m^−2^. (**d**) Photographs show the mass evolution of ice with (**left**) and without (**right**) the hierarchically designed CA film. (**e**) Mass decay of the ice with and without the hierarchically designed film. (**f**) Protection for snow [105]. (**g**) Schematic illustration of the outdoor radiative cooling setup. Inset: optical photograph of set-up. (**h**) Fruit preservation using CPHS aerogel [106].

In the context of ice and snow protection applications, several non-phase-separation fabrication methods have also demonstrated significant potential. For example, Yue et al. [30] developed a dielectric/polymer composite film with excellent radiative cooling performance. By combining a low-cost polymer matrix with inorganic dielectric particles via a phase separation method, they fabricated a hierarchically porous composite (HPC) membrane with micro/nanostructures and tunable spectral properties. The HPC film exhibited a solar reflectance of 98.26% and an infrared emissivity of 97.56%, achieving a sub-ambient cooling temperature of 9.1 °C under a solar irradiance of 890 W/m^2^ and an effective radiative cooling power of 87.2 W/m^2^. As shown in Figure 11a–f, Li et al. [105] fabricated a hierarchical radiative cooling film based on environmentally friendly cellulose acetate molecules. This film achieved passive protection against ice masses of varying forms and sizes and demonstrated the capability to effectively slow the melting rate of local glaciers.

In related research, Fan et al. [106] prepared a thermally insulating dry gel based on cellulose nanocrystals. The developed dry gel exhibits a low thermal conductivity of 0.0671 W·m^−1^·K^−1^, a high solar reflectance of approximately 92.52%, and a high mid-infrared emissivity of about 94.45%. As shown in Figure 11g, the outdoor radiative cooling performance of the dry gel material was systematically studied under clear-sky conditions in Yantai, Shandong Province (37°37′05″ N, 121°7′50″ E). The results demonstrated efficient cooling effects in both fruit preservation and ice storage applications, as illustrated in Figure 11h.

Shi et al. [107] developed a porous thermoplastic polyurethane film doped with MgO (PTM membrane), which integrates radiative cooling (temperature reduction) and broad-spectrum antibacterial functions into a single material, achieving a synergistic preservation strategy combining “physical cooling + biological bacteriostasis.” In strawberry preservation experiments, the material enabled localized cooling of 4–8 °C, effectively inhibiting enzymatic browning and bacterial growth, and maintained optimal freshness of the strawberries for at least 5 days. Zhang et al. [102] prepared a cellulose acetate/zinc oxide (CA/ZnO) nanocomposite film via a water-assisted phase separation method. Under strong solar irradiation, the material achieved a daytime temperature reduction of 13.8 °C. In strawberry preservation tests, it extended the storage time of strawberries to 9 days, outperforming other commercially available food packaging materials. The entire preservation process operates with zero energy consumption, relying solely on the passive radiative cooling mechanism, which aligns with the sustainability requirements of the food cold chain.

In the field of food preservation, fabrication methods beyond phase separation have also yielded promising results. For instance, Xu et al. [103] synthesized a hydrogel based on a radiation/evaporation synergistic effect, designed to protect fruits from solar radiation and thermal stress. The hydrogel achieved a solar reflectance of 0.89 and an atmospheric window emissivity of 0.90, enabling daytime radiative cooling. In a fruit preservation experiment, the quality attributes of pears (*Pyrus sinkiangensis*) stored in a preservation box were analyzed after 2 h of sunlight exposure. Although preliminary studies have confirmed the potential of radiative cooling films in retarding ice melt and preserving food, current research remains largely focused on simple overlay experiments demonstrating cooling effects. There has been limited exploration into specific, large-scale applications such as customized packaging for batch production. To fully realize the application potential of these films in ice preservation and food storage, future work should expand the scope of analysis to include varied food types, storage conditions, and material performance optimization. Developing efficient, environmentally friendly, and practically viable radiative cooling film solutions will require deeper investigation into scalable designs and real-world implementation pathways.

## 6. Conclusions and Outlook

This review systematically presents the fundamental principles of radiative cooling, the classification system of phase separation methods, and the design strategies and application prospects of porous radiative cooling films fabricated via phase separation techniques. By focusing on phase separation as a specific fabrication paradigm, we elucidate the core advantage of this technical pathway. Through precise regulation of the thermodynamic equilibrium and phase inversion kinetics of the polymer-solvent-nonsolvent system, it is possible to directly program the multi-scale porous architecture for the materials. This intrinsic control over pore morphology, size distribution, and hierarchical structure serves as the physical foundation for achieving tailored spectral emissivity and scattering properties, representing the fundamental characteristic that distinguishes the phase separation method from other fabrication routes.

Specifically, this work demonstrates that phase separation is not merely a preparation process but rather a structural design paradigm for material optical functionality. Techniques such as non-solvent-induced phase separation and vapor-induced phase separation enable the directed construction of porous films with specific photothermal response characteristics by adjusting formulation parameters and environmental conditions. The in situ incorporation of nanoparticles during the phase separation process further provides an interfacial engineering strategy for synergistically enhancing solar reflectance and mid-infrared emission. Currently, the field has progressed from initial proof-of-concept stages to performance optimization and scalable exploration for practical applications. Films fabricated using the phase separation method have demonstrated clear energy-saving potential in scenarios such as building energy efficiency and personal thermal management. However, phase-separation-derived radiative cooling films still face several challenges and limitations:

1. Efficiency constraints under real-world conditions

Radiative cooling efficiency remains subject to various practical limitations. Daytime cooling performance is significantly influenced by environmental factors such as air humidity, wind speed, and cloud cover. Outside the atmospheric window, cooling devices absorb additional energy due to solar radiation and weather conditions like clouds and haze. Furthermore, the energy radiated by cooling devices is limited by their own temperature constraints. Although ideal radiative efficiency continues to improve, real-world performance substantially lags theoretical potential due to atmospheric and climatic influences. Thus, further research is needed to enhance temperature adaptability and optimize performance for diverse climatic conditions.

2. Environmental considerations

The environmental and non-toxic characteristics of radiative cooling films are other critical factors. Adopting eco-friendly materials, ensuring the biocompatibility and potential biodegradability of these films, and minimizing pollution and health risks (such as water-based coatings [108], cellulose [23], or bio-based alternatives [109]) throughout the manufacturing process, are essential to achieving a truly sustainable and green cooling solution.

3. Balancing functionality and aesthetics

To meet the demands of various application scenarios, radiative cooling films must be available in different colors and appearances. However, incorporating colorants may compromise cooling performance. Achieving vibrant coloration while maintaining high radiative cooling efficiency presents a significant challenge.

4. Standardization and system integration

The superior performance of a standalone radiative cooling device does not directly translate to its effectiveness within practical building energy systems. As demonstrated in the research by Zhou et al. [110], the authors modeled the integration of water-based radiative cooling panels with heat exchangers and cold storage systems. This quantification of the impact of system design parameters—such as water flow rate, panel size, and operational strategies—is crucial for achieving substantial indoor air temperature reductions (~12.7 °C) and assessing overall economic viability. The future development of radiative cooling technology must shift from pursuing the ultimate optical performance of individual materials toward establishing a design, testing, and integration methodology aimed at systemic energy efficiency and cost-effectiveness. This entails developing more universal performance evaluation standards and advancing systematic engineering research that accounts for specific climatic conditions and end-use energy demands.

5. Modeling accuracy and theoretical evolution

Currently, some radiative cooling studies still employ simplified atmospheric emissivity models (such as constant values or cosine law approximations) in theoretical modeling. These models fail to adequately capture the complex radiative transfer characteristics of the real atmosphere. To more accurately evaluate and predict the performance of materials and devices, it is essential to incorporate more sophisticated atmospheric radiation models (e.g., MODTRAN, LBLRTM) and comprehensively account for the vertical stratification effects of atmospheric parameters such as temperature and humidity. Advancing the transition from schematic principles to engineering precision represents a critical direction for enhancing the reliability of design optimization and performance prediction.

In summary, although phase-separation-derived radiative cooling films offer a promising pathway for sustainable cooling applications, current challenges must be addressed. These include enhancing environmental adaptability, reducing costs, integrating aesthetic design, establishing unified standards, and advancing the refinement of theoretical models. Addressing these aspects will be crucial for promoting the broader practical implementation of this technology.

## Figures and Tables

**Table 1:** Principles, driving forces, and applicable materials of different phase separation methods.

Method	Principle	Driving Force	Applicable Materials
TIPS	A homogeneous solution of a polymer and high-boiling-point solvent is prepared at a high temperature, and liquid–liquid phase separation is induced by cooling	Temperature variation	Hardly soluble polymers
NIPS	The mutual diffusion between the solvent and the non-solvent leads to phase separation and solidification of the polymer solution	Solvent/non-solvent exchange	Most soluble polymers
VIPS	The penetration of the non-solvent (vapor) and the evaporation of the solvent jointly induce phase separation and solidification of the solution	Vapor infiltration	Systems sensitive to coagulation bath shock
EIPS	Non-solvent droplets precipitate and serve as pore-forming templates.	Solvent evaporation	Polymers in stable mixed solvent systems
RIPS	The increase in molecular weight or changes in polarity cause thermodynamic instability in the system, leading to phase separation.	Polymerization chemical reaction	Cross-linked polymer network porous materials
PIPS	A homogeneous mixture of photosensitive monomers/prepolymers and a porogen undergoes phase separation initiated by photopolymerization under light irradiation.	Photochemical reaction	Photocurable monomers/prepolymers containing porogens

**Table 2:** Commonly used solvents and their solubility related parameters.

Solvent	Dispersion Parameter	Polarity Parameters	Hydrogen Bond Parameters	Total Solubility Parameter	Boiling Point (°C)
DMAc [60]	16.8	11.5	10.2	22.7	165
DMF [60]	17.4	13.7	11.3	24.8	153
DMSO [61]	18.4	16.4	10.2	26.7	189
HMPA [62]	18	12.3	7.2	22.9	202
TMU [63]	16.8	8.2	11.1	21.7	176.5
TMP [64]	16.8	6	10.2	22.3	197.2
Ac [65]	15.5	10.4	7.0	20.0	57.1
THF [66]	16.8	5.7	8.0	19.4	65

## Data Availability

The original contributions presented in this study are included in the article. Further inquiries can be directed to the corresponding authors.
